# Antibodies against influenza A (H7N9) virus among veterinarians in China before 2013

**DOI:** 10.1111/irv.12288

**Published:** 2014-10-29

**Authors:** Xiuchen Yin, Jie Tao, Fanglong Zhao, Baizhong Rao, Huili Liu

**Affiliations:** a Institute of Animal Science and Veterinary Medicine, Shanghai Academy of Agricultural SciencesShanghai, China; b Shanghai Municipal Key Laboratory of Agri-genetics and BreedingsShanghai, China

To the editor:

A recent outbreak of a novel avian-origin reassortant influenza A (H7N9) virus in eastern China has raised great concerns owing to the rapidly progressive lower respiratory tract infections in infected individuals. As of August 1, 2014, a total of 151 humans infections of H7N9 (resulting in 45 deaths) in China have been reported to the World Health Organization.^1^ Indeed, inclusion of a wide range of high-risk groups often provides a better understanding of immunogenic responses to a novel influenza virus infection. A previous serologic study (conducted by Yuelong Shu and colleagues) did not find any evidence for human infection with the novel avian-origin influenza A (H7N9) virus in poultry workers before 2013 in southeastern China.^2^ The other serologic study conducted in Zhejiang province found that 741 healthcare and non-healthcare workers did not have subclinical or asymptomatic A (H7N9) infection and suggested that more extensive serological investigation of asymptomatic or subclinical A (H7N9) infection among different high-risk groups in various regions should be performed in China.^3^

Of critical importance in this regard is the fact that occupational groups studied have not included veterinarians. Epidemiological and serological evidence have already indicated that poultry farm and live market workers are high-risk groups.^2^,^4^ However, due to the nature of their work environment and practices, veterinarians are also at high risk of exposure to avian influenza virus and may have more intense and frequent exposure to diseased poultry than poultry workers themselves.^4^,^5^

To assess whether subclinical human infection with the novel H7N9 virus occurred in the other poultry exposure group (veterinarians) before 2013, we collected single serum samples anonymously from practicing veterinarians (*n* = 820: 288 from Shanghai, 192 from Anhui, 199 from Zhejiang, and 141 from Gangsu) during December 2011 to December 2012 (Table 1). We employed a hemagglutination inhibition (HI) assay according to the WHO instructions.^6^ Our HI assay used horse erythrocytes instead of chicken red blood cells (RBCs), to increase the assay sensitivity. An H7 antigen A/pigeon/Shanghai/S1421/2013(H7N9) derived from the emergent H7N9 AIV was obtained from the Chinese Academy of Agricultural Sciences (CAAS), Harbin Veterinary Research Institute (Harbin, China). A serum sample was considered positive when the HI titer ≥80.^3^ Sera from the veterinarians were also studied for antibodies against influenza A (H7N9) virus using a new competitive ELISA kit that targets H7 hemagglutinin antigen (Immune Technology, Suzhou, China).^7^ Negative control serum and positive control serum specimens were included in each plate to provide a diverse range of controls.

**Table 1:** Characteristics of participants in the serological survey in China before 2013

		No. demographic description		
Locations	No.	Median of age (range)	No. of female (%)	Median of occupation age at time of poultry contact
Shanghai	288	37 (23–63)	31 (10·76)	17·33
Anhui	192	43 (29–66)	20 (10·42)	20·37
Zhejiang	199	34 (24–53)	15 (7·58)	13·56
Gansu	141	41 (21–60)	12 (8·51)	14·48

None of the samples were positive for H7 AIV infection by HA-specific ELISA and HI assays using ≥80 cut-off antibody titer. Although HI titers of 20 were detected in 19 of 820 serum samples, the lower 40 titers might have resulted from cross-reactivity with previous human influenza virus infections. None of the veterinarians had evidence of previous infection with the emergent H7N9 AIV before 2013.

Our serologic study first found no evidence for human infection with the novel avian-origin influenza A (H7N9) virus in veterinarians before 2013 in China. To our knowledge, this is the first investigation of seroprevalence of Influenza A (H7N9) in occupationally exposed veterinarians in eastern China. A better understanding of interspecies transmission of avian influenza is a crucial component in efforts to minimize the effects of the next pandemic. First, high-risk groups with frequent and close contact to infected avians may be among the first to be infected and, by spreading the illness to their families and communities, may serve as a bridging population to the general population. Second, different to the other high-risk groups, the veterinary with frequent and close contact to other infected animals. As none-avian animals such as pigs and dogs in China have been shown to be infected with AIVs,^8^,^7^,^9^ seasonal influenza vaccination is recommended for veterinary, to reduce the risk of joint infections with human and avian viruses and consequent risk of virus reassortment or recombination. As novel viruses may rapidly change, it seems prudent to continue surveillance for the H7N9 virus among high-risk groups such as poultry workers, veterinarians, healthcare and non-healthcare workers.
